# Discovery of cell-type specific DNA motif grammar in cis-regulatory elements using random Forest

**DOI:** 10.1186/s12864-017-4340-z

**Published:** 2018-01-19

**Authors:** Xin Wang, Peijie Lin, Joshua W. K. Ho

**Affiliations:** 1 Victor Chang Cardiac Research Institute, Darlinghurst, NSW 2010 Australia; 2 St. Vincent’s Clinical School, University of New South Wales, Darlinghurst, NSW 2010 Australia

**Keywords:** DNA motif, Transcription factor, Random Forest, Cell-type specificity, Cis-regulatory element

## Abstract

**Background:**

It has been observed that many transcription factors (TFs) can bind to different genomic loci depending on the cell type in which a TF is expressed in, even though the individual TF usually binds to the same core motif in different cell types. How a TF can bind to the genome in such a highly cell-type specific manner, is a critical research question. One hypothesis is that a TF requires co-binding of different TFs in different cell types. If this is the case, it may be possible to observe different combinations of TF motifs – a motif grammar – located at the TF binding sites in different cell types. In this study, we develop a bioinformatics method to systematically identify DNA motifs in TF binding sites across multiple cell types based on published ChIP-seq data, and address two questions: (1) can we build a machine learning classifier to predict cell-type specificity based on motif combinations alone, and (2) can we extract meaningful cell-type specific motif grammars from this classifier model.

**Results:**

We present a Random Forest (RF) based approach to build a multi-class classifier to predict the cell-type specificity of a TF binding site given its motif content. We applied this RF classifier to two published ChIP-seq datasets of TF (TCF7L2 and MAX) across multiple cell types. Using cross-validation, we show that motif combinations alone are indeed predictive of cell types. Furthermore, we present a rule mining approach to extract the most discriminatory rules in the RF classifier, thus allowing us to discover the underlying cell-type specific motif grammar.

**Conclusions:**

Our bioinformatics analysis supports the hypothesis that combinatorial TF motif patterns are cell-type specific.

**Electronic supplementary material:**

The online version of this article (10.1186/s12864-017-4340-z) contains supplementary material, which is available to authorized users.

## Background

Transcription factors (TFs) are proteins which usually bind to genomic DNA at specific DNA sequences (motifs) [1]. The binding of different TFs to DNA is critical for the regulation of gene expression in almost all important biological processes, including embryogenesis [2–4], cell cycle and development control [5–7], and response to intercellular signals and environment [8, 9]. We already know different TFs can be added as exogenous reprogramming factors to convert somatic cells to other cell types (e.g., fibroblasts to pluripotent cells) [10]. One interesting observation is that many TFs bind to genomic DNA at different loci depending on the cell-type and biological context (such as signalling pathway activation) in which the TF is expressed, even though the TF binds to the same core motif across different cell types and conditions. For example, Frietze et al. found that the same TF (TCF7L2) can bind to different genomic loci across different cell types [11]. One hypothesis is that any particular TF requires the co-binding of different combinations of cell-type specific TFs, such as master regulators [12], in different cell-types [13]. If this is the case, it may be possible to observe different combination of motifs – a motif grammar – being present in the binding sites of the same TF across different cell types.

Several studies have attempted to explore this hypothesis using a computational synthetic biology approach [14, 15]. Furthermore, spatial co-occurrence patterns of specific pairs of motifs have also been systematically investigated computationally [16]. Some other studies have investigated the questions of cell-type specificity using a combination of sequence features, chromatin structure as well as histone modification marks [17, 18]. These works all lend evidence to the assumption that a DNA motif grammar – a set of rules based on combinations of TF motifs – in cis-regulatory module exists, and is cell-type specific.

To further test this motif grammar hypothesis, we wish to explore the use of a machine learning classifier to predict the cell-type specificity of a TF binding site. Several published studies have used a similar machine learning approach to explore cell-type specificity, including a SVM classification model using *k*-mer sequences in TF ChIP-seq peaks, histone modifications and DNase accessibility data as features to train and predict cell-type specific TF binding sites [19]; a computational approach that considers cell-type specific histone H3K27ac DNA profiles around transcription start sites with neural network to predict gene expression in mouse embryonic stem cells [20]; and a deep neural network approach to predict the sequence specificities of DNA and RNA-binding proteins [21].

Nonetheless, we wish to explore a specific question that more directly tests the motif grammar hypothesis – can we build a multi-class machine learning classifier based on combinations of sequence motifs alone? Furthermore, we note that many machine learning classifiers that have good performance (e.g., SVM, deep neural network) are hard to visualise and interpret. In other words, it is often difficult to extract the rules – the grammar – from these machine learning models. In this work, we propose to address these issues by developing a Random Forest (RF) based multi-class cell-type classifier based on TF motif combinations alone, and use a recently developed rule-mining approach to extract important discriminatory rules from the trained RF classifier.

Random Forest, first published as random decision forests [22], is a machine learning algorithm for classification and regression. A RF consists of a collection of decision trees, where each tree consists of a random subsample of features [23]. Random Forest could measure feature importance by calculating the ‘mean decrease accuracy’. Compared to classification based on an individual decision tree, a RF has been shown to be more robust against the overfitting problem [24]. With the ability to perform multi-class classification, and generally having superior performance, RF is widely used in various fields of biological and biomedical research [25–29]. One short-coming of RF is that it is commonly considered to be a ‘black box’ machine learning method, as it is not easy to extract and visualise the decision rules that lead to a particular prediction. Recently, some methods have been developed to extract interpretable rules from a RF [30], and software tools have been developed to extract, trim and prune importance rules in a RF [31].

In the following study, we use this RF approach to analyse two published ChIP-seq TF datasets (TCF7L2 and MAX) from ENCODE [32]. Our finding reveals that combinatorial DNA motifs can be cell-type specific, and that we can extract biologically meaningful motif grammars from a RF classifier.

## Results

### Identification of cell-type specific cis-regulatory elements

The TF ChIP-seq datasets we tested by RF were downloaded from the ENCODE project dataset [32] (see Additional file 1). Data for TF proteins TCF7L2 (Transcription factor 7-like 2) and MAX (myc-associated factor X) were chosen because they were systematically profiled across a good number of cell-types (6 cell types in TCF7L2 and 5 cell types in MAX). TCF7L2, which at a downstream effector of the Wnt singling pathway, is a TF that affects the transcription of a variety of genes that affect a diverse set of biological functions [33, 34]. It is also linked to human diseases, including type 2 diabetes [35–37] and a variety of cancers [33, 38, 39]. MAX is a TF protein that is able to form homo-dimers or hetero-dimers with other proteins, which include MYC, MXL1 and MAD [40]; these dimers promote cell differentiation and apoptosis [40]. Many reports also showed that MAX is related to the small cell lung cancer (SCLC) [41–43].

For the TCF7L2 dataset, there are six human cell-lines, including colon cancer cells (HCT116), embryonic kidney cells (HEK293), cervical carcinoma cells (HeLa-S3), liver cancer cells (HepG2), mammary gland adenocarcinoma cells (MCF-7) and pancreatic cancer cells (PANC-1). For the MAX dataset, the five human cell-lines are adenocarcinomic alveolar basal epithelial cells (A549), lymphoblastoid cells (GM12878), immortalised myelogenous leukaemia cells (K562), HeLa-S3 and HepG2.

**Fig. 1:**
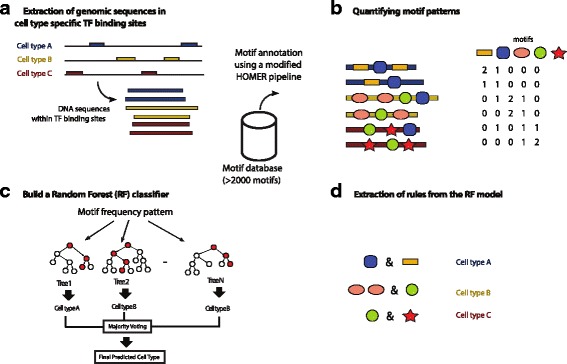
Our bioinformatics workflow for DNA motif annotation, Random Forest (RF) classifier training and motif grammar extraction. The workflow consists of four steps. **a** Step 1: extraction of the genomic sequences from the cell-type specific TF binding sites. **b** Step 2: annotation of these sequences using a large database of motifs. **c** Step 3: training of a RF classifier. **d** Step 4: Motif rule (grammar) extraction from the RF classifier

We extracted the 500 strongest unique ChIP-seq peaks by *p*-value from each cell-line in both the TCF7L2 and MAX datasets (see Additional file 2). We have developed a pipeline to extract the DNA sequences at these ChIP-seq peaks, and identified known motifs (based on a large motif database from ENCODE [44]) in these sites using a DNA motif annotation pipeline (see Methods). We were able to extract the number of occurrences of each motif in these sites. This becomes the training set for our RF classifier. The predictive ability of our RF using the TCF7L2 and MAX datasets is evaluated using cross-validation. Furthermore, the final trained RF classifier is mined to extract meaningful rules. Figure 1 illustrates the workflow of our DNA motif annotation and RF analysis pipeline.

### Combinations of motifs are predictive of cell-types

We applied 10 times 10-fold Cross-Validation (CV) to evaluate the ability of RF to classify TF sites from different cell-types based on motif occurrence patterns. In order to determine the optimal size of binding site in the two datasets, we first investigate the effect of varying the size of the binding site from +/− 5 bp around the peak centre to +/− 300 bp around the peak centre. We found that the best prediction accuracy can be achieved when the TF binding site is ~240 bp in length (120 bp up and downstream from the peak centre; see Additional file 3). Therefore**,**we use 240 bp around the centre of ChIP-seq peaks as our TF binding sites for all downstream analyses.

**Fig. 2:**
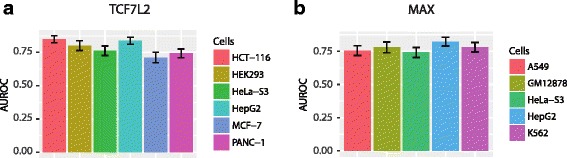
The Area Under the Receiver Operator Characteristics Curve (AUROC) of the RF classifiers based on Cross-Validation. **a** The TCF7L2 dataset. **b** The MAX dataset. The error bars indicate standard deviations

We then use 10 times 10-fold CV to evaluate the predictive ability of a RF classifiers trained on the TCF7L2 and the MAX datasets. Using the Area Under the Receiver Operating Characteristic curve (AUROC) as a measure, we found that our RF classifiers indeed has a clear ability to discriminate among the 6 cell types profiled in the TCF7L2 dataset, and among the 5 cell types profiled in the MAX dataset (Fig. 2).

To further test if the RF algorithm can distinguish different cell types based on the combinations of motifs in the 240 bp region of the ChIP-seq peaks from the same antibody, we applied our pipeline on six additional TF datasets, each consisting of TF binding sites from five to seven cell lines (see Additional file 1). Cross-validation analysis reveals that we can use motif combinations to build a RF classifier with a reasonable sensitivity and specificity (see Additional file 4), further supporting our hypothesis that TF motif combinations are predictive of cell-type specificity.

### Meaningful cell-type specific motif grammars can be extracted from a RF classifier

**Fig. 3:**
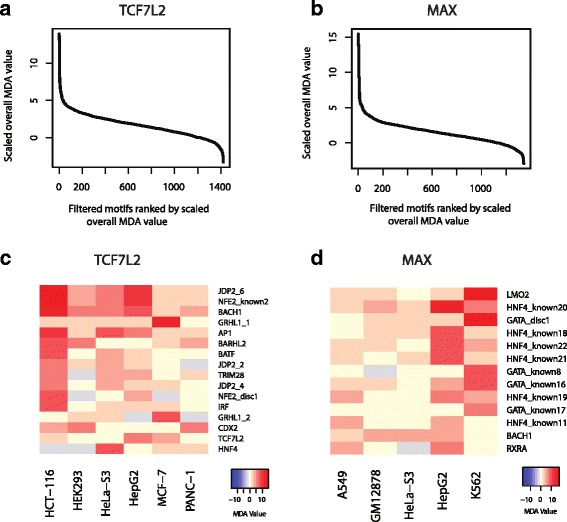
The mean decrease accuracies (MDA) of motif importance extracted from the trained RF classifiers**. a** The sorted MDA values extracted from the TCF7L2 RF. **b** The sorted MDA values extracted from the MAX RF. **c** Heat map showing the MDA values of the top motifs in the TCF7L2 RF. **d** Heat map showing the MDA values of the top motifs in the MAX RF

To identify the important features (motifs) in a trained RF classifier, we extracted the mean decrease accuracies (MDA [45]) values of all the motifs based on the RFs trained using the TCF7L2 and the MAX datasets (Fig. 3). The MDA, output by the *randomForest* package, informs the overall importance of a motif in the RF model. More specifically, the MDA value of a variable represents the increase in out-of-bag error that is caused by removing that variable. The MDA values reported in Fig. 3 is scaled by the standard deviation of the MDA values of all the variables. A positive MDA value indicates that inclusion of that variable is important in the RF classifier, whereas a negative MDA value indicates that inclusion of that variable negatively impact the accuracy of the RF classifier. In both TF datasets, there are a small number of motifs that have a high MDA values, suggesting that most of the discriminatory power comes from a small number of motifs (Fig. 3a and b). Using those motifs with high scaled mean decrease accuracies (with scaled MDA greater than 6), we can extract the list of motifs that are cell-type specific in the two datasets (Fig. 3c and d). Only about a dozen motifs have high MDA. This finding indicates that although many motifs are present around the +/− 120 bp region of a ChIP-seq peak centre, only a small proportion of motifs show cell-type specificity. This distribution also leads to our interest to extract motif grammar rules from the RF classifiers.

### Discovery of motif grammar rules from random forests

As RF has the ability to distinguish cell types based on motif occurrence in TF binding sites, we next attempt to extract interpretable rules from the trained RF classifiers. In particular, we use a rule mining approach that is implemented in an R package called inTrees [31], which can extract, measure, prune and select rules from a RF. The algorithm of inTrees is able to extract all the frequent decision rules from all the trees in an RF, and select set of most highly predictive and non-redundant rules based on all the training data [31]. By using the *inTrees* package, a number of cell-type specific rules were extracted from the two RF classifiers (Tables 1 and 2). Most of these rules are based on motifs with a high MDA value (e.g., NFE2 and BACH1 in the TCF7L2 dataset; HNF4, AP1, LMO2 and GATA in the MAX dataset), thus supporting that inTrees are discovering important rules that are present in the RF. We found that the majority of the TFs in the extracted rule have reported cell-type or tissue-specific expression specificities, such as IRF in lymphoblastoid cell-line [46, 47], AP-1 in cervical cancer [48, 49], HNF4 in liver and colon cells [50], LMO2 [51] and GATA in leukaemia cells [52, 53]. For some other motifs in the table, their TF proteins are considered as oncogenes or related to tumorigenesis (e.g., JDP2 [54], BACH1 [55] and NFE2 [56, 57]). The motif of GRHL1, whose TF is reported to interact with estrogen receptor, is also found to be expressed in mammary gland adenocarcinoma cells [58]. Our results suggest that our rule mining approach can indeed discover biologically meaningful motif rules from a RF classifier.

**Table 1:** DNA motif rules extracted from the RF classified trained on the TCF7L2 dataset

Rule	Prediction	Reference
NFE2 > =3	HCT-116 (colon cancer cells)	[56, 57]
GSX1 < =35 & NFE2 > =2		NFE2 [56, 57]
BACH1 > =2 & FOXO3 < =2 & HOMEZ < =5		BACH1 [55]
BACH1 < =1 & E2F > =2 & HOXB13 > =3	HEK293 (embryonic kidney cells)	E2F [63], HOXB13 [64]
CDX2 > =5 & GATA > =2 & MAF = 0		GATA [65, 66]
BACH1 < =1 & HNF4 < =6 & HOXA13 > =3 & OTX1 > =9		HOXB13 [64]
JDP2 > =2 & SOX21 > =37	HeLa-S3 (cervical carcinoma cells)	JDP2 [54]
HNF4 > =2	HepG2 (liver cancer cells)	[50]
HOXB13 < =3 & JDP2 < =1 & TCF7L2 > =5		TCF7L2 [67]
HNF4 > =1 & HOXC10 < =16 & SOX9 > =5		HNF4 [50], SOX9 [68]
GRHL1 > =4	MCF-7 (mammary gland adenocarcinoma cells)	GRHL1 [58]
No rule identified	PANC-1 (pancreatic cancer cells)	

The numbers in the rules represent the motif frequency detected in the +/− 120 bp regions from the peak centre

**Table 2:** DNA motif rules extracted from the RF classified trained on the MAX dataset

Rule	Prediction	Reference
IRF > =1	GM12878 (lymphoblastoid cells)	[46, 47]
JDP2 > =1	HeLa-S3 (cervical carcinoma cells)	[54]
AP1 > =9 & HESX1 > =2 & LMO2 > =2		AP1 [48, 49]
EMX1 < =11 & ETS < =12 & HNF4 > =1	HepG2 (liver cancer cells)	HNF4 [50], ETS [69]
HNF4 > =1 & IRF4 < =4 & RUNX2 < =4 & TAL1 < =4		HNF4 [50]
ALX3 < =26 & EVX1 > =6 & GATA > =2 & LMO2 > =2	K562 (immortalised myelogenous leukaemia cells)	GATA [52, 53], LMO2 [51]
GATA > = 4 & HNF4 = 0 & POU4F3 < =4		GATA [52, 53]
No rule identified	A549 (adenocarcinomic alveolar basal epithelial cells)	

The numbers in the rules represent the motif frequency detected in the +/− 120 bp regions from the peak centre

## Discussion

Using two ENCODE TF ChIP-seq datasets, our study shows that different combination of a small number of motifs is sufficient to discriminate TF binding sites that are used in different cell types. Also, we demonstrate how we could use a Random Forest (RF) classifier for classification and rule extraction, highlighting the power of opening up a ‘black box’ machine learning model.

Our pipeline is unique as we are annotating TF binding site sequences with >2000 known motifs. The use of such as comprehensive TF motif database is important as the goal is to test the hypothesis that motif combinations alone can be predictive of cell-type-specific TF binding sites (cis regulatory elements). Nonetheless, our study is only the first step towards deciphering the DNA motif grammar. Besides motif combinations, cell-type specificity may also be affected by the spatial arrangement of the motifs, existing histone modifications and DNA accessibility [13, 18, 19], and long range interactions [59]. Nonetheless, our finding is an important step towards discovering a cell-type specific TF motif grammar.

## Conclusion

Our bioinformatics analysis supports the hypothesis that combinatorial TF motif patterns are cell-type specific.

## Methods

### Datasets

We downloaded ChIP-seq peak files of two TFs TCF7L2 and MAX from the ENCODE ChIP-seq Experiment Matrix [60]. To maintain consistency across datasets, we only used the ChIP-seq peaks from ENCODE/SYDH standard (mapped to hg19 reference genome) by peak caller “PeakSeq1.0”. For multiple entries in the ENCODE database, only IgG normalised ChIP-seq peaks were chosen.

### Motif annotation and random Forest implementation

We used the R package *randomForest* [61] for the implementation of RF. We systematically evaluated how many trees are needed to train a good RF based on our two datasets. The default tree number 500 is adequate for stabilising the out of bag (OOB) error (see Additional file 5). Therefore 500 trees were used in all our analyses. The input features for the Random Forest are the top 500 strongest unique ChIP-seq peaks by *p*-value for each of the six cell-lines in the TCF7L2 and each of the five cell-lines in MAX datasets. Therefore, the input for each RF is a matrix of number of occurrence of 2067 motifs in 3000 (or 2500) cell-type specific TF binding sites in the TCF7L2 dataset (or the MAX dataset). Our motif database consists of 2065 motifs from ENCODE [44], as well as two de novo TCF7L2 motifs identified by Frietze et al. [11]. The motif database was then converted to the HOMER motif database format.

### Evaluation of classifier performance

We have assessed the performance of the RF classifier through several methods, namely cross validation, out of bag errors and ROC curves. Cross-validations were used to estimate the classifier errors. We have performed 10-fold cross-validations ten times on Amazon AWS using the R packages *foreach*, *doMC* and *caret*. Out of bag errors make use of the unselected samples in each tree in the forest to estimate the classifier errors, and have been shown accurate empirically [62]. Besides estimating classifier errors, we have also calculated AUROC values to assess the performance of the RF classifiers. More specifically, a binary classifier for each cell-line can be obtained as follows: for each sample, the RF classifier outputs the percentage of decision trees that predict each cell-line, and this percentage is used as the discrimination threshold of a binary classifier for the cell-line. The AUROC values for each of these binary classifiers are shown in Fig. 2.

### Identification of cell-type discriminatory motifs in a RF classifier

Two independent methods were used to identify cell-type discriminatory motifs. Firstly, the mean decrease accuracy (MDA), output by the *randomForest* package, informs the overall importance of a motif in the RF model. Specifically, the RF training was performed in two rounds. In the first round, features with negative MDAs were removed so that these irrelevant features were not present in the final random forest. Then, we optimised the number of features in each decision tree using the ‘tuneRF’ function in the *randomForest* package by minimizing the out-of-bag errors. More specifically, through out-of-bag error estimation, it estimates the loss of accuracy after randomly permuting the values of each motif. We then plot the MDA of all the motifs in Fig. 3.

A second method is to extract frequent and important rules present in the decision trees in an RF, using the R package *inTrees*. For this procedure, we used RFs trained from the two whole datasets as the inputs. Rules were extracted and pruned using the ‘getRuleMetric’ and ‘pruneRule’ functions respectively. Then a set of relevant and non-redundant rules were selected using regularised RFs through the ‘selectRuleRRF’ function, after which the rules were further selected based on the frequency and error – rules with a frequency below 8% or error above 0.7 were eliminated. The resulting selected rules are included in Tables 1 and 2.

## Additional files

Additional file 1: Table S1. Datasets used in this study. The ENCODE data we used in this study with the information of TFs, cell-lines, reference genome, peak caller information and the GEO Accession ID. (XLS 42 kb)

Additional file 2: Figure S1. –log10 *p*-value distribution of the peaks on the TCF7L2 and the MAX datasets. The positions of the 500th peak in each plot ranked by p-value were highlighted in red lines. (PDF 343 kb)

Additional file 3: Figure S2. – Cross validation of RF classifiers trained on the TCF7L2 and the MAX datasets. We employed F1 score (F1 = 2 * precision * recall / (precision + recall)) to measure the performance of the RF classifier for each cell line. We evaluated the discriminatory power of TF binding sites that are +/− 5 bp to +/− 300 bp from the centre of each of the TF ChIP-seq peak. (PDF 29 kb)

Additional file 4: Figure S3. – The Area Under the Receiver Operator Characteristics Curve (AUROC) of the RF classifiers based on cross-validation. (A) The CEBPB dataset. (B) The CHD2 dataset. (C) The EP300 dataset. (D) The JUND dataset. (E) The MXI1 dataset. (F) The RAD21 dataset. Error bars indicate standard deviations. (PDF 157 kb)

Additional file 5: Figure S4. – Out of Bag (OOB) curves of the RF of TCF7L2 dataset and MAX dataset. From the curve of the OOB error rate against the number of trees, we find that 500 trees are sufficient to minimise errors, and increasing the number of trees would not help the RF classifier to perform better. We used 500 trees in our downstream analyses. (PDF 18 kb)

## Abbreviations

AUROC: Area under Receiver Operating Characteristic curve
ChIP-seq: Chromatin Immuno-Precipitation Sequencing
CV: Cross-Validation
ENCODE: Encyclopedia of DNA Elements
HOMER: Hypergeometric Optimization of Motif EnRichment
MAX: Myc-associated factor X
MDA: Mean Decrease Accuracy
RF: Random Forest
ROC curve: Receiver Operating Characteristic curve
TCF7L2: Transcription factor 7-like 2
TF: Transcription Factor
