# Supplementary material for: Discovery of cell-type specific DNA motif grammar in cis-regulatory elements using random Forest
Source: BMC Genomics. 2018 Jan 19;19(Suppl 1):929. doi: 10.1186/s12864-017-4340-z (PMC5780765; doi:10.1186/s12864-017-4340-z)

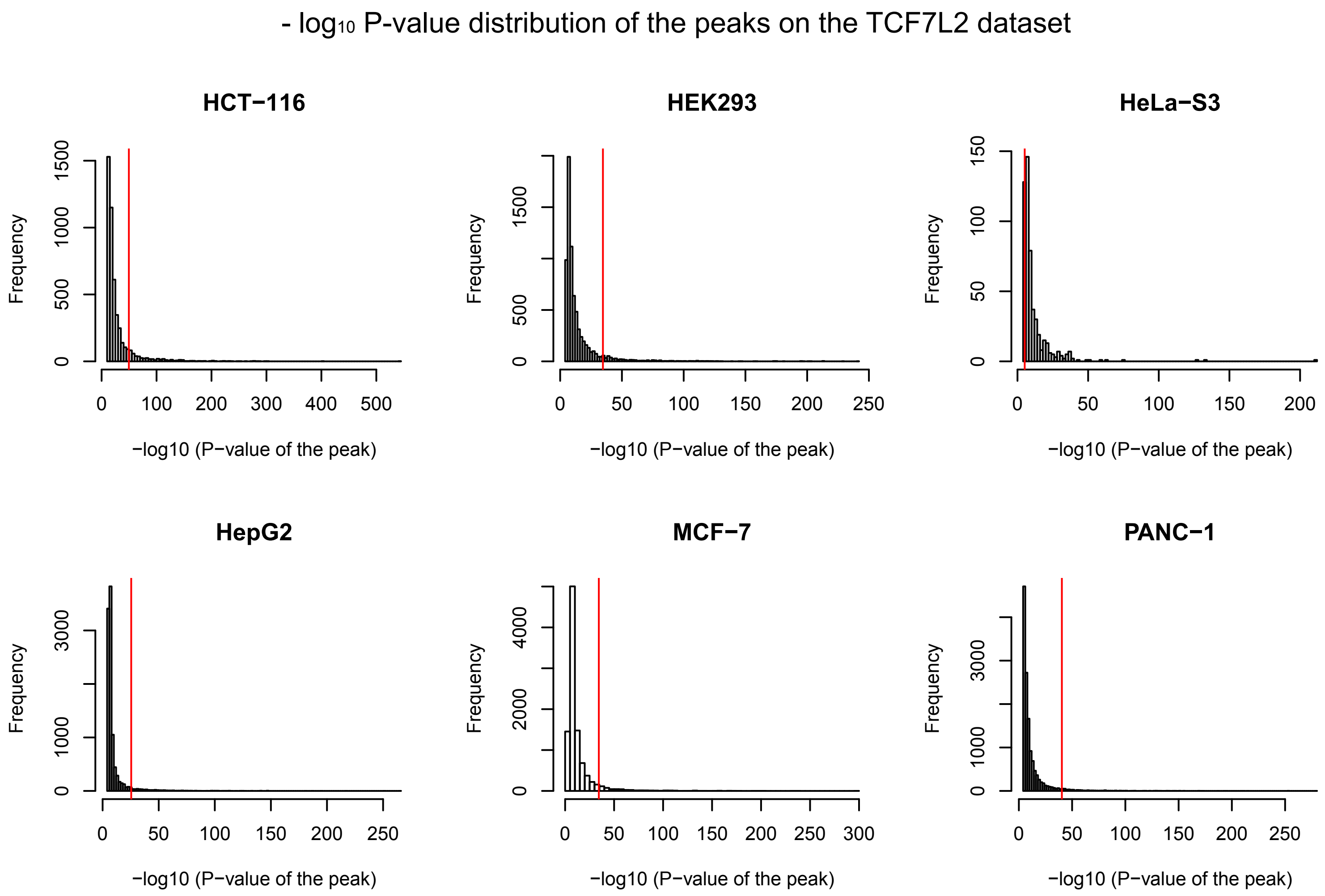

- log10 P-value distribution of the peaks on the TCF7L2 dataset
HCT−116
HEK293
HeLa−S3
HepG2
MCF−7
PANC−1
Frequency
−log10 (P−value of the peak)

- $\log_{10}$ P-value distribution of the peaks on the MAX dataset

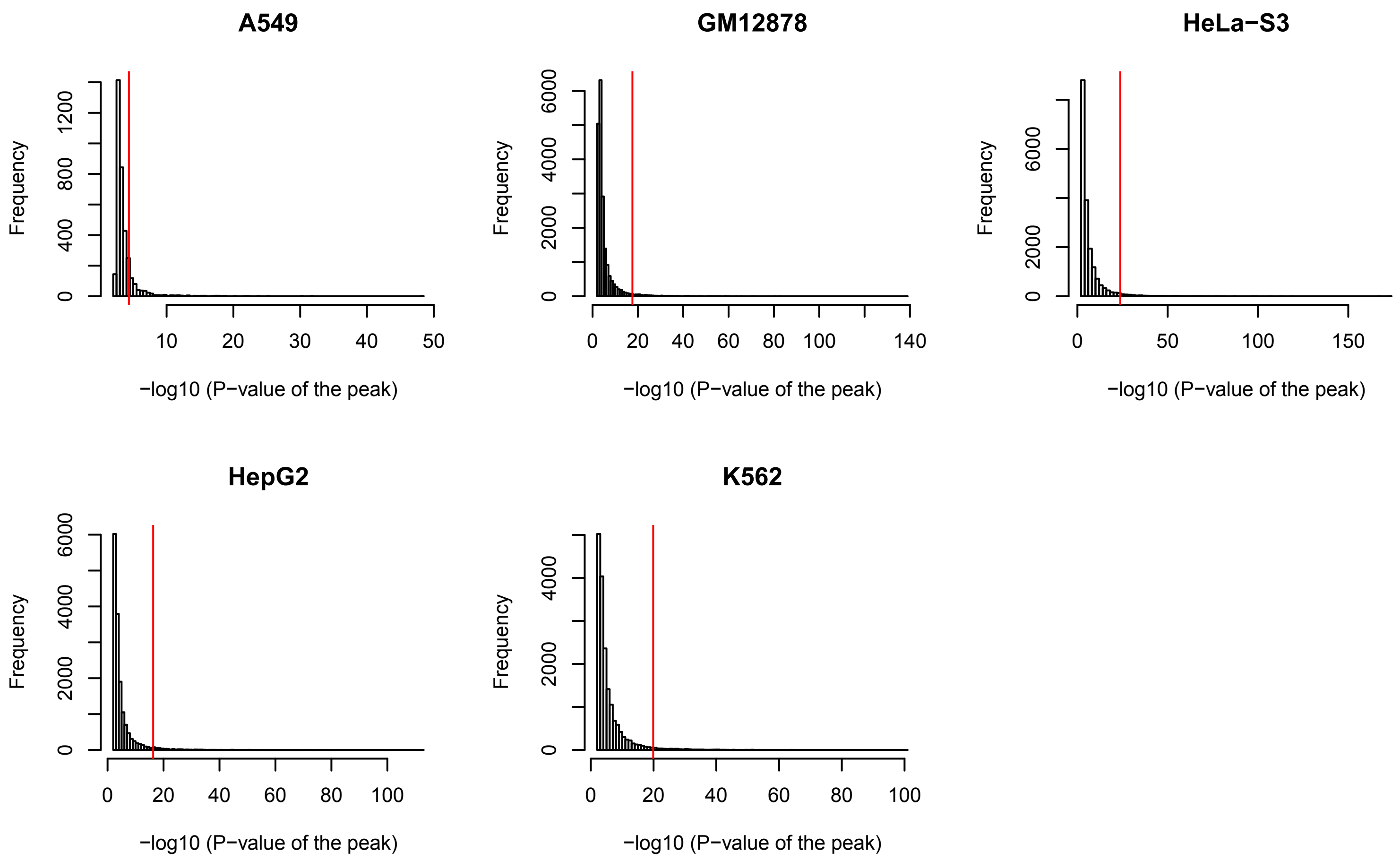

Supplement: Supplementary file 2 — –log10 p-value distribution of the peaks on the TCF7L2 and the MAX datasets. The positions of the 500th peak in each plot ranked by p-value were highlighted in red lines. (PDF 343 kb) [file 12864_2017_4340_MOESM2_ESM.pdf]
