# Supplementary material for: Discovery of cell-type specific DNA motif grammar in cis-regulatory elements using random Forest
Source: BMC Genomics. 2018 Jan 19;19(Suppl 1):929. doi: 10.1186/s12864-017-4340-z (PMC5780765; doi:10.1186/s12864-017-4340-z)
Supplement: Supplementary file 3 — – Cross validation of RF classifiers trained on the TCF7L2 and the MAX datasets. We employed F1 score (F1 = 2 * precision * recall / (precision + recall)) to measure the performance of the RF classifier for each cell line. We evaluated the discriminatory power of TF binding sites that are +/− 5 bp to +/− 300 bp from the centre of each of the TF ChIP-seq peak. (PDF 29 kb) [file 12864_2017_4340_MOESM3_ESM.pdf]

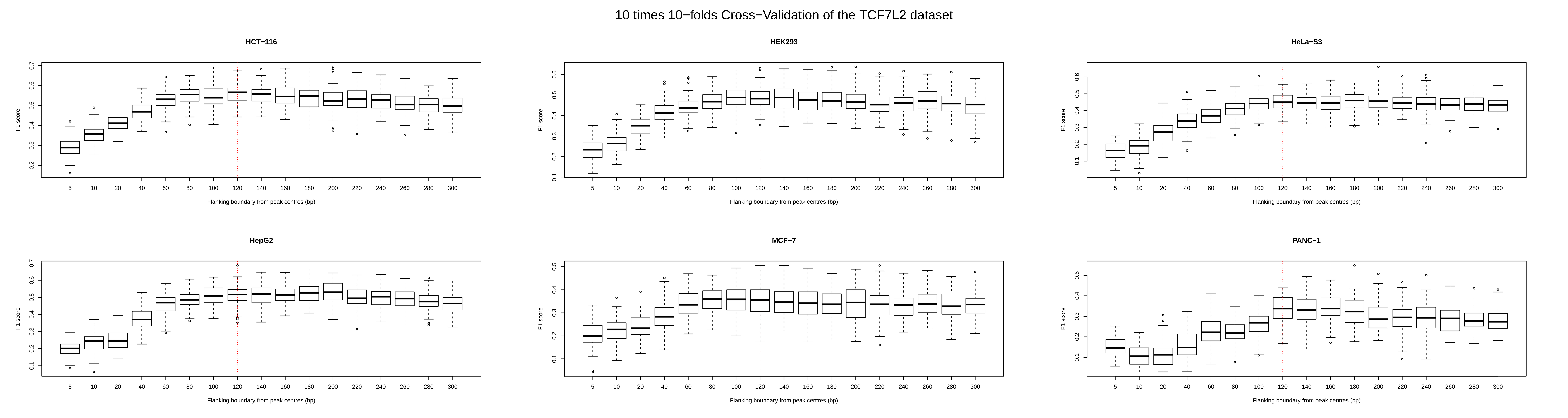

10 times 10−folds Cross−Validation of the TCF7L2 dataset
HCT−116
HEK293
HeLa−S3
HepG2
MCF−7
PANC−1
F1 score
Flanking boundary from peak centres (bp)
5 10 20 40 60 80 100 120 140 160 180 200 220 240 260 280 300

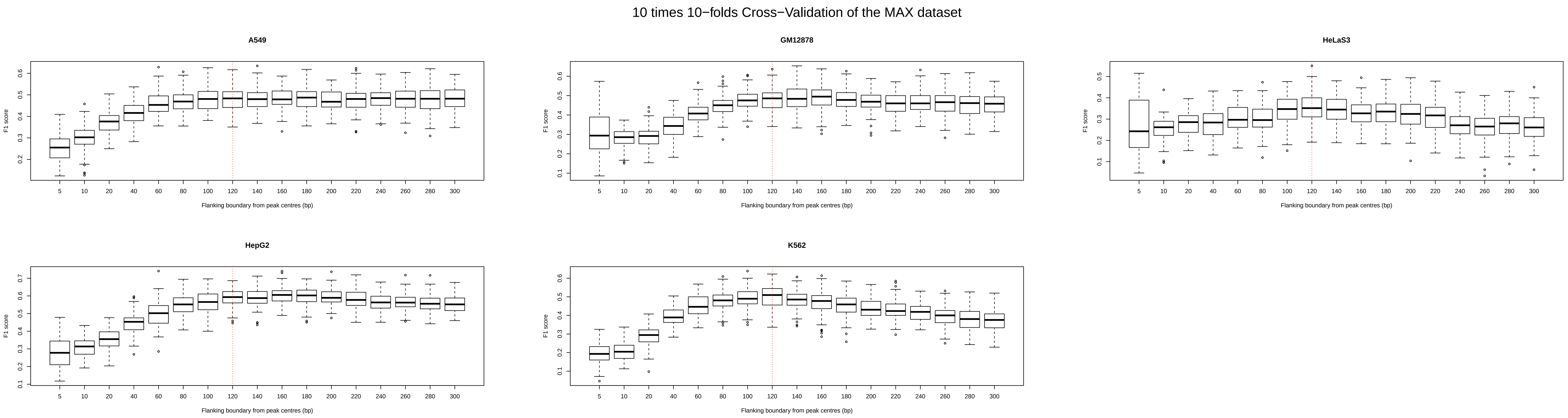

10 times 10−folds Cross−Validation of the MAX dataset
A549
GM12878
HeLaS3
HepG2
K562
F1 score
Flanking boundary from peak centres (bp)
5 10 20 40 60 80 100 120 140 160 180 200 220 240 260 280 300
