# Supplementary figures and images for: Discovery of cell-type specific DNA motif grammar in cis-regulatory elements using random Forest
Source: BMC Genomics. 2018 Jan 19;19(Suppl 1):929. doi: 10.1186/s12864-017-4340-z (PMC5780765; doi:10.1186/s12864-017-4340-z)

## The AUROC of the RF classifier based on Cross-Validation

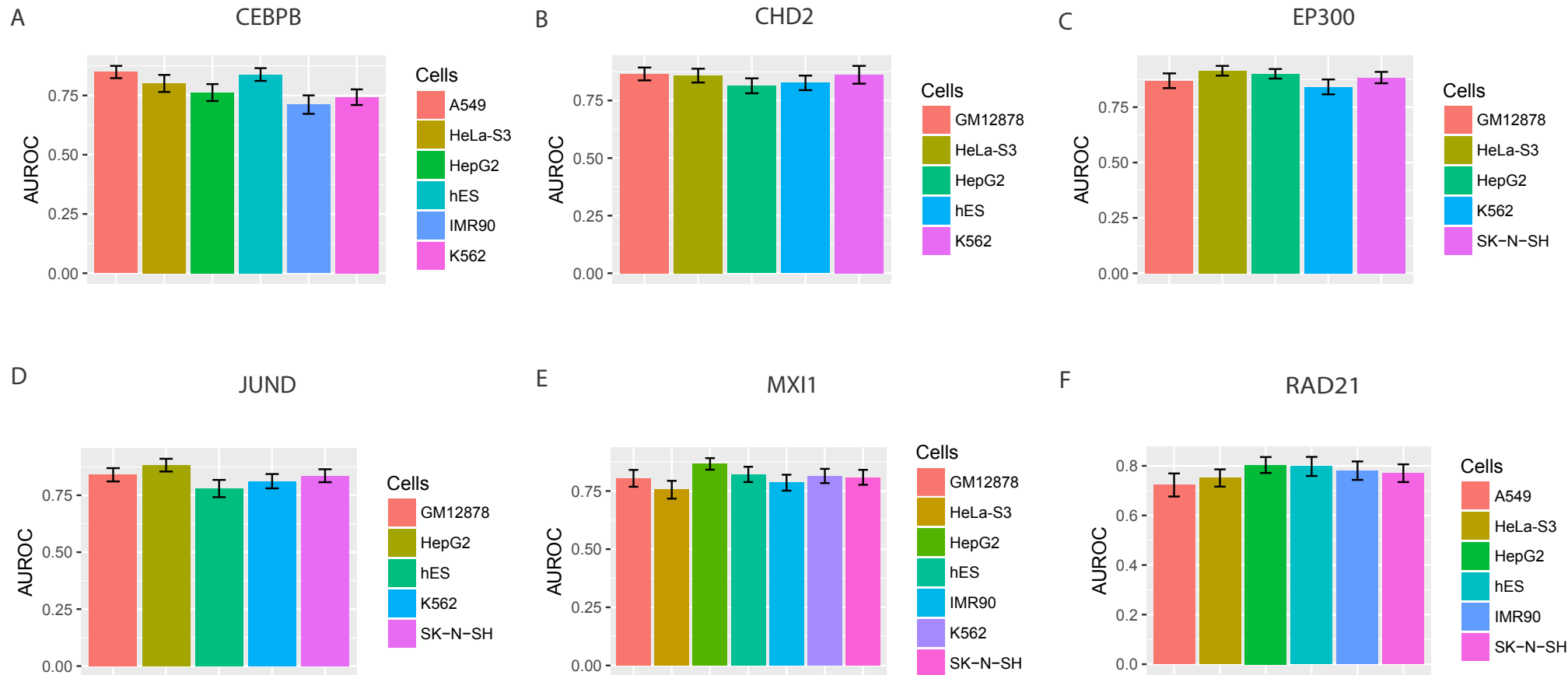

Supplement: Supplementary file 4 — – The Area Under the Receiver Operator Characteristics Curve (AUROC) of the RF classifiers based on cross-validation. (A) The CEBPB dataset. (B) The CHD2 dataset. (C) The EP300 dataset. (D) The JUND dataset. (E) The MXI1 dataset. (F) The RAD21 dataset. Error bars indicate standard deviations. (PDF 157 kb) [file 12864_2017_4340_MOESM4_ESM.pdf]

**Out of Bag (OOB) evaluation of RF of TCF7L2 dataset**

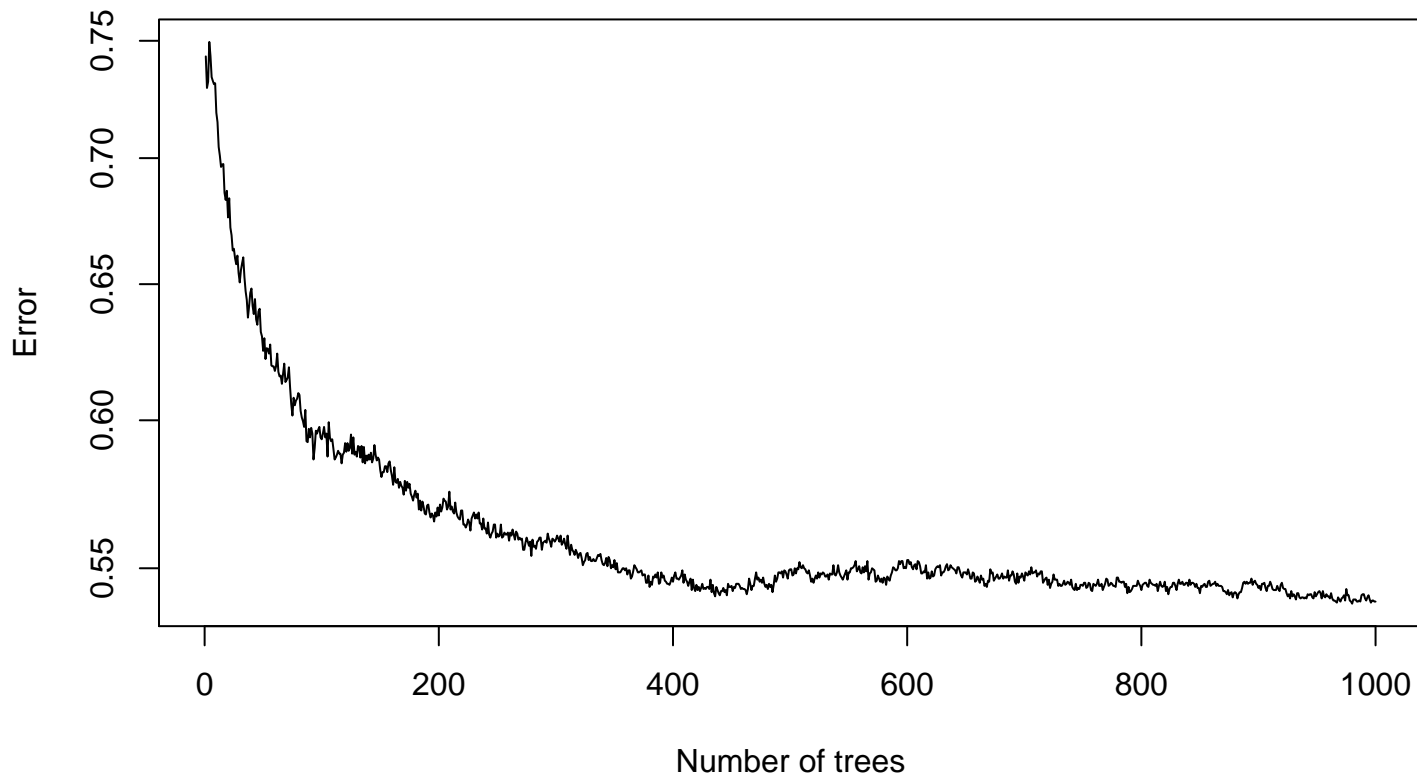

**Out of Bag (OOB) evaluation of RF of MAX dataset**

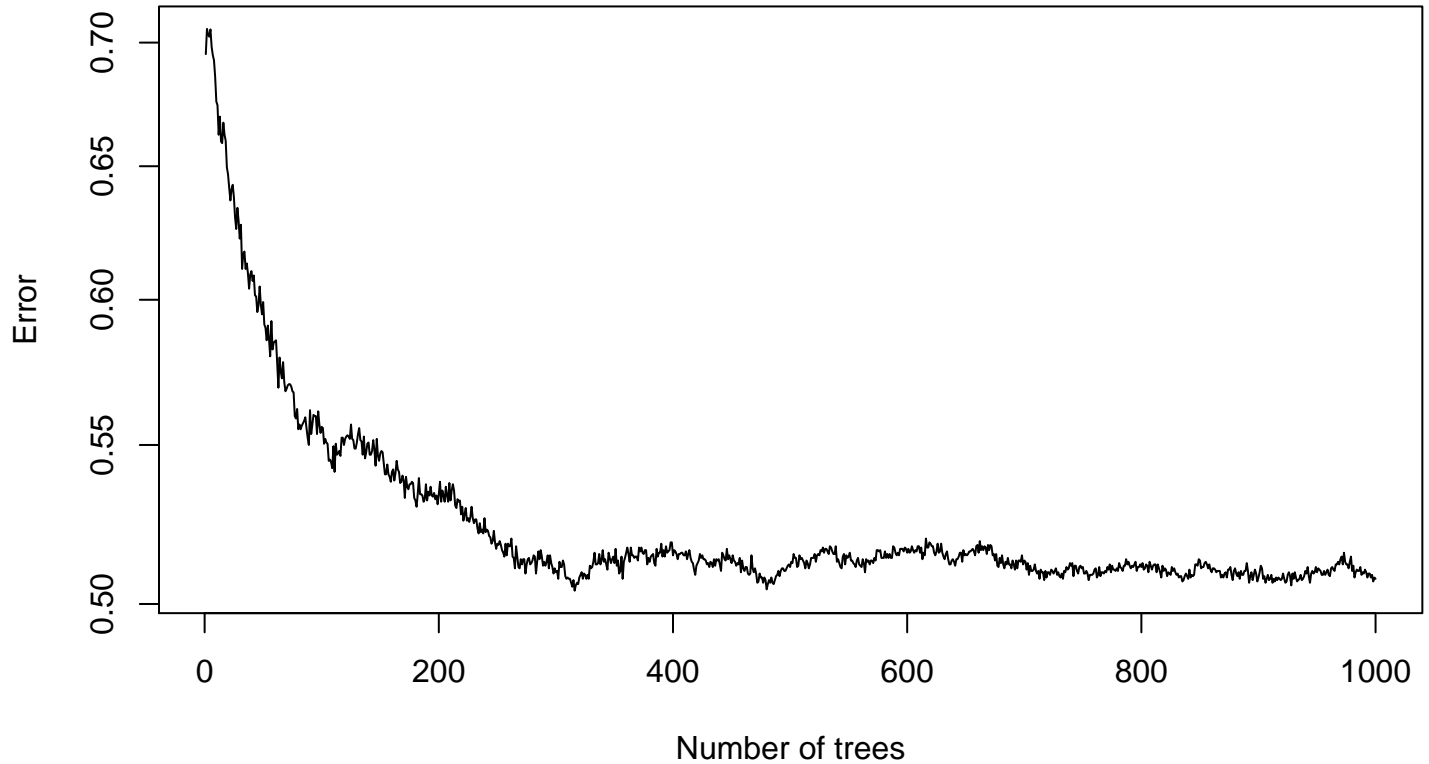

Supplement: Supplementary file 5 — – Out of Bag (OOB) curves of the RF of TCF7L2 dataset and MAX dataset. From the curve of the OOB error rate against the number of trees, we find that 500 trees are sufficient to minimise errors, and increasing the number of trees would not help the RF classifier to perform better. We used 500 trees in our downstream analyses. (PDF 18 kb) [file 12864_2017_4340_MOESM5_ESM.pdf]
